# Caprine Monocytes Release Extracellular Traps against *Neospora caninum In Vitro*

**DOI:** 10.3389/fimmu.2017.02016

**Published:** 2018-01-19

**Authors:** Zhengtao Yang, Zhengkai Wei, Carlos Hermosilla, Anja Taubert, Xuexiu He, Xiaocen Wang, Pengtao Gong, Jianhua Li, Xichen Zhang

**Affiliations:** ^1^College of Basic Medical Sciences, Jilin University, Changchun, China; ^2^College of Veterinary Medicine, Jilin University, Changchun, China; ^3^Faculty of Veterinary Medicine, Institute of Parasitology, Justus Liebig University Giessen, Giessen, Germany

**Keywords:** *Neospora caninum*, caprine, monocytes, extracellular traps, apicomplexa

## Abstract

*Neospora caninum* is an obligate intracellular apicomplexan parasite that causes reproductive loss and severe economic losses in dairy and goat industry. In the present study, we aim to investigate the effects of *N. caninum* tachyzoites on the release of extracellular traps (ETs) in caprine monocytes and furthermore elucidated parts of its molecular mechanisms. *N. caninum* tachyzoite-induced monocytes-derived ETs formation was detected by scanning electron microscopy. H3 and myeloperoxidase (MPO) within monocyte-ETs structures were examined using laser scanning confocal microscopy analyses. The results showed that *N. caninum* tachyzoites were not only able to trigger ETs formation in caprine monocytes, but also that monocyte-released ETs were capable of entrapping viable tachyzoites. Histones and MPO were found to be decorating the DNA within the monocytes derived-ETs structures thus proving the classical components of ETs. Furthermore, inhibitors of NADPH oxidase-, MPO-, ERK 1/2-, or p38 MAPK-signaling pathway significantly decreased *N. caninum* tachyzoite-triggered caprine monocyte-derived ETosis. This is the first report of ETs release extruded from caprine monocytes after *N. caninum* exposure and thus showing that this early innate immune effector mechanism might be relevant during the acute phase of caprine neosporosis.

## Introduction

*Neospora caninum* is an obligate intracellular parasite that naturally infects a wide host range, such as dogs, cattle, sheep, and caprines (1–3). Neosporosis is considered as a major cause for reproductive disorders and thereby causing severe economic losses in cattle (4, 5). More recently, vast amount of data have been generated suggesting the key role of *N. caninum* as major pathogen of the caprine reproductive tract thereby causing mummification and abortion in primary infected animals (6, 7). Although drugs, such as sulfonamides and pyrimethamine, for treatment of neosporosis are available (8), new efficient options for control and treatment of this disease remain to be further investigated.

In recent years, adaptive immunity-related research has clarified the cellular immune response of intermediate hosts, such as caprines, against this parasite and improved our better understanding of resulting pathogenesis of ruminant neosporosis. During primary *N. caninum* infection, both natural killer cells and CD8^+^ T cell, together with CD4^+^ T cells have been demonstrated to play a pivotal role in producing interferon gamma (IFN-γ) (9). Additionally, IFN-γ and interleukin 17 (IL-17) can also been secreted by native T cells in contact with *N. caninum-*infected macrophages (10). Meanwhile, Th1-released cytokines may be regulated by Th2-derived cytokines, such as IL-10, IL-4, and transforming growth factor beta to allow the improvement of the materno-fetal immunity in order to avoid fetal rejection by these abortive parasites (11, 12). This balance between Th1- and Th2-released cytokines may provide necessary environment for the activation of host cellular adaptive immune response against *N. caninum* tachyzoites.

The release of extracellular traps (ETs) has been recognized as a novel effector mechanism against pathogens in several types of innate immune cells, such as polymorphonuclear neutrophils (PMNs), eosinophils, macrophages, mast cells, and monocytes (13–15). ETs are formed during a cell death process, known as ETosis, and mainly composed of DNA backbone fibers, histones, myeloperoxidase (MPO), neutrophil elastase (NE), cathelicidin, and so on. In previous studies, the critical role of monocyte-triggered ETs in host innate immune response against *Besnoitia besnoiti* tachyzoites has been described (16). In addition, monocytes are significantly increased in the blood samples of *N. caninum*-seropositive cows after 180 days of gestation (17). And during the first days of infection, monocytes have been recruited by excreted/secreted antigens from *N. caninum* to the sites of infection, which will further promote the process of parasite invasion and proliferation (18). These results suggest the vital role of monocytes in innate immune response against *N. caninum* infection. However, effects of *N. caninum* on the formation of ETs in caprine monocytes have not been explored so far. In the present study, we investigated the effects of *N. caninum* tachyzoites on the release of caprine monocytes ETs and furthermore intended to elucidate some of its molecular components as well as on the signaling pathways being involved in monocyte ETosis.

## Materials and Methods

### *N. caninum* Tachyzoites *In Vitro* Culture

The tachyzoites of *N. caninum* (strain Nc-1) were maintained in VERO cells monolayers at 37°C/5% CO_2_. The detail conditions of *N. caninum* tachyzoites culture and isolation was following to our previous study (19).

### Isolation of Caprine Monocytes

Adult healthy caprines (*n* = 3, 2 years old) were bled by puncture of the femoral vein and blood was collected. The caprine monocytes were isolated according to the caprine monocyte isolation kit^®^ (Tian Jin Hao Yang Biological Manufacture Co., China). In brief, 5 ml of heparinized blood was applied on the top of separating gradient medium in sterile 15 ml centrifugation tubes. After centrifugation (500 *g*, 25 min, 4°C), caprine monocytes were collected and red blood cells mixed with monocytes were thereafter lysed by lysis buffer (Tian Jin Hao Yang Biological Manufacture Co., China). Thereafter, the monocytes were washed twice (500 *g*, 5 min, 4°C) and resuspended in serum-free RPMI 1640 medium (Hyclone, USA). After purification, monocytes were counted and cultured in glass coverslips, contained in 24-well-tissue culture plates or 96-well-tissue culture plates for ET-related experiments. All animal experiments were approved by Laboratory Animal Welfare of Jilin University.

### Scanning Electron Microscopy (SEM)

Caprine monocytes were cocultured with viable *N. caninum* tachyzoite (ratio 1:2) for 60 min and 90 min. The samples were fixed in 4.0% glutaraldehyde, washed twice with sterile PBS for 60 min, and postfixed in 1.0% osmium tetroxide (Merck) for 40 min. After three time washings with distilled water, the samples were dehydrated in ascending ethanol concentrations, frozen in tertiary butyl alcohol at −20°C and sputtered with gold. The samples were examined by SEM (Hitachi S-3400N, Japan).

### Fluorescence Microscopy Analysis

Caprine-monocytes were seeded onto coverslides allocated in 24-well tissue culture plates and stimulated with vital *N. caninum* tachyzoites (ratio: 1:1) for 90 min. The samples were fixed with 4% (w/v) paraformaldehyde (MPO, 15 min) or cold methanol (histone, 15 min) on poly-l-lysine-coated glass coverslips, permeabilized with 0.1% Triton X-100 and blocked for 2 h at room temperature. The specific antibodies: anti-MPO antibody (Orb16003; Biorbyt), antihistone antibody (LS-C353149; Life Span BioSciences, Inc.) were used for detection of MPO and H3 on monocyte-derived ETs structures. The antirabbit IgG-FITC conjugated was purchased from Bioworld Technology Inc. The samples were then counterstained with 5 μM Sytox Orange for 10 min and observed by scanning confocal microscope (Olympus FluoView FV1000).

### Quantitation of Monocyte-Derived ETs

The formation of caprine monocyte-derived ETs was quantified using Sytox Green (Invitrogen). In brief, caprine monocytes were seeded in 96-well plate and stimulated with *N. caninum* tachyzoites for 30, 60, or 90 min. In parallel settings, the cells were pretreated with the following inhibitors: the NADPH oxidase inhibitor (DPI, Sigma-Aldrich), the MPO inhibitor (ABAH, Calbiochem), the inhibitors of ERK1/2-signaling pathway (UO126, Sigma) and P38 MAPK-signaling pathway (AB202190, Sigma-Aldrich). The activities of ERK 1/2- and p38 MAPK signaling pathway was also determined by western blot analysis. Then, samples were coincubated with Sytox Green (Invitrogen) at concentration of 5 µM for 10 min, and examined by spectrofluorometric analysis (488 nm excitation/523 nm emission wavelength) using a fluorometric plate reader Infiniti M200 (TECAN, Austria).

### Detection of Reactive Oxygen Species (ROS)

Reactive oxygen species production in *N. caninum* tachyzoites-stimulated caprine monocytes was determined by 2,7 dichlorofluorescein diacetate (DCFH-DA, Sigma). Briefly, caprine monocytes were incubated with DCFH-DA (10 µM, 15 min) prior to the stimulation with vital *N. caninum* tachyzoite (ratio: 1:3 or 1:6, 180 min, 37°C). Monocytes stimulated with zymosan (1 mg/ml, Sigma-Aldrich) served as positive controls. Unstimulated monocytes cultured in plain medium alone served as negative controls. In parallel settings, the cells were pretreated with the NADPH oxidase inhibitor (DPI, Sigma-Aldrich) for 30 min before *N. caninum* stimulation. Finally, the samples were washed three times with phenol red-free RPMI 1640 medium and measured by using a fluorometric plate reader Infiniti M200 (TECAN, Austria) and flow cytometry at 488 nm excitation/525 nm emission wavelength.

### Detection of Lactate Dehydrogenase (LDH) Activities

For detection of LDH activities, freshly isolated caprine monocytes were stimulated with viable *N. caninum* tachyzoites (ratio: 1:1) for 30, 60, 90, and 120 min at 37°C in 96-well tissue culture plates. After incubation, the plates were centrifuged at 300 *g* for 5 min and the LDH activity in the supernatant was determined by the LDH Cytotoxicity Assay kit^®^ (Beyotime Biotechnology, China). The positive control was examined according to the manufacturer’s protocols.

## Results

### Tachyzoites of *N. caninum* Exposed to Caprine Monocytes Trigger ET Formation

**Figure 1:**
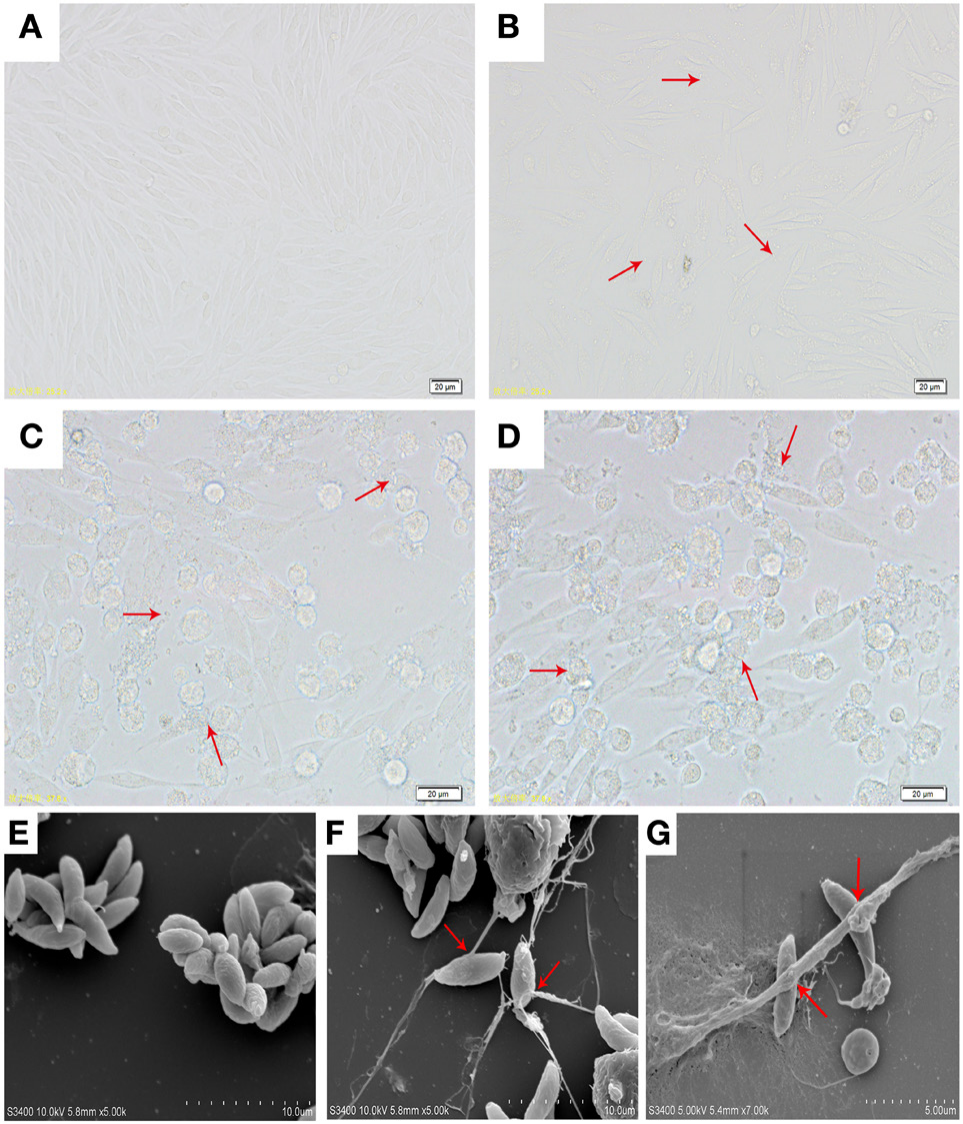
**(A)** Optical microscopy of VERO cells morphology. **(B)** Optical microscopy of VERO cells infected with *N. caninum* tachyzoite for 3 h. **(C,D)** Optical microscopy of VERO cells infected with *N. caninum* tachyzoite for 48 h. **(E)** *N. caninum* tachyzoite. **(F,G)** Extracellular traps were formed by caprine monocytes, and *N. caninum* tachyzoite was captured in these monocytes network structures. Three independent experiments were carried out. Red arrows in **(B–D)** showed *N. caninum* tachyzoites. Red arrows in **(F,G)** showed caprine monocyte-ETs triggered by *N. caninum* tachyzoites.

**Figure 2:**
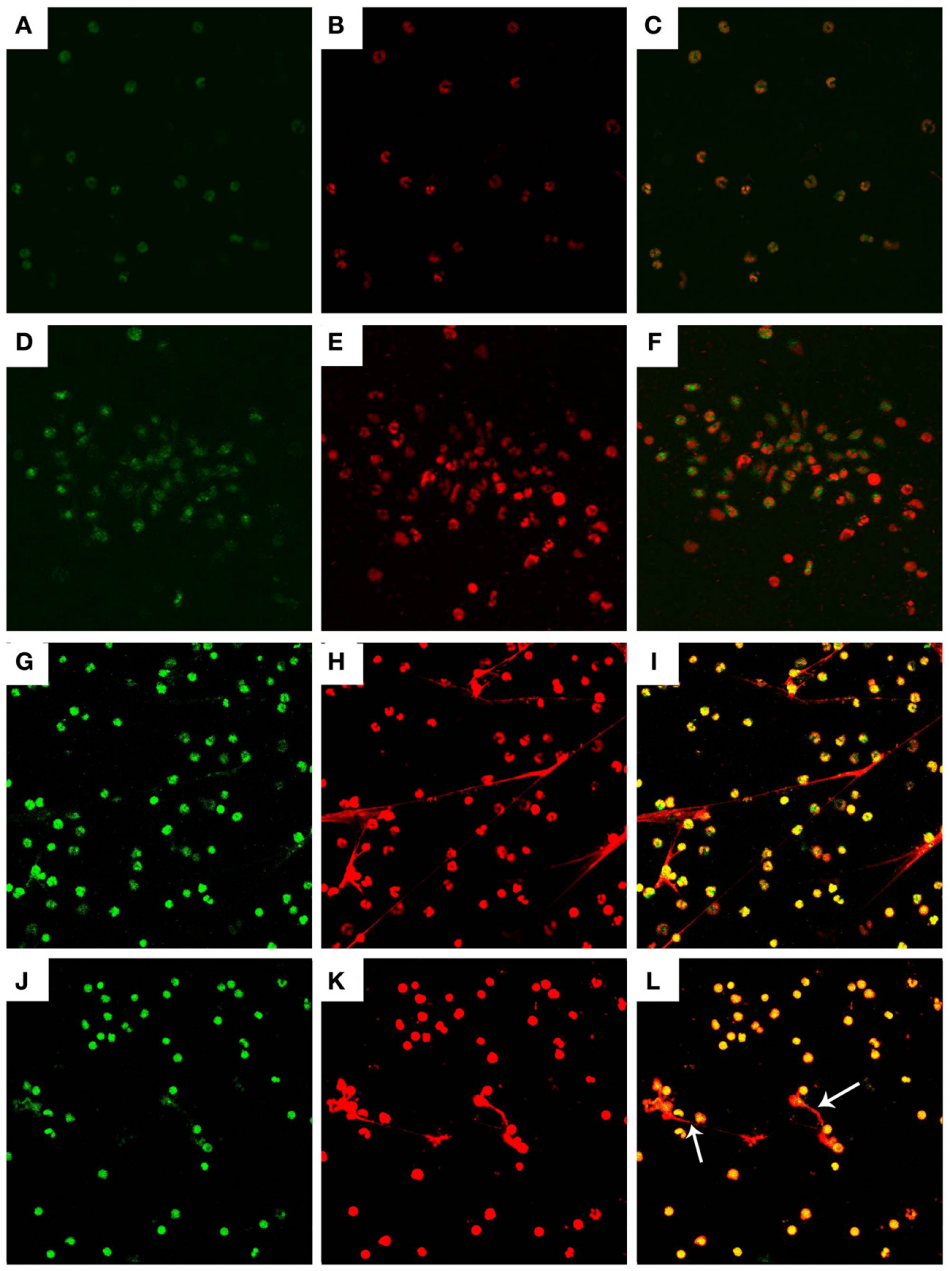
Histone and myeloperoxidase (MPO) in *Neospora caninum* tachyzoite-triggered caprine monocyte-ETs structures. Caprine monocytes were stimulated with *N. caninum* tachyzoite (ratio: 1:1) for 90 min. Detection of DNA decorated with of H3 and MPO within the monocyte-ETs structures were examined using a laser scanning confocal microscope. **(A)** Control histone (green). **(G)** Histone (green) in monocyte-ETs structures. **(D)** Control MPO (green). **(J)** MPO (Green) in monocyte-ETs structures. **(B,E)** Control DNA (red). **(H,K)** DNA within these network structures was stained with Sytox Orange (red). **(C,F,I,L)** Respective merge of DNA decorated with histone and MPO. Three independent experiments were carried out. White arrows showed caprine monocyte-ETs structures triggered by *N. caninum*.

The microscopy image of the VERO cell growth and the infectivity of *Neopsora caninum* tachyzoites are shown in Figures 1A–D. Freshly isolated vital *N. caninum* tachyzoites obtained from infected *in vitro* VERO cell cultures seemed to be vital as demonstrated by their gliding motility as well as typical morphological features of apicomplexan protozoan tachyzoites (see Figure 1E). Moreover, SEM analysis revealed that the exposure of *N. caninum* tachyzoites to caprine monocytes resulted in the formation of a delicate network of thicker and thinner strands of fibers originating from monocytes and being firmly attached to the parasite surface, seemingly entrapping them (Figures 1F,G). In addition, *N. caninum* tachyzoite-induced ETs were confirmed by fluorescence microscopy analyses (Figures 2I,L). There results clearly suggest that *N. caninum* tachyzoites are potent inducers of ET formation in exposed caprine monocytes.

### H3 and MPO in *N. caninum-*Triggered Caprine Monocyte-ETs

Detection of the DNA backbone decorated with H3 and MPO in monocyte-ETs structures were examined using laser scanning confocal microscopy analyses. Control groups of histone (Figure 2A) and MPO (Figure 2D) were colocated with DNA (Figures 2B,E) in respective merge images (Figures 2C,F). Extracellular DNA within these network structures was stained with Sytox Orange (Figures 2H,K). Fluorescence imaging analyses further revealed colocalization of DNA decorated with H3 (Figure 2G) and MPO (Figure 2J) in *N. caninum* tachyzoite-triggered caprine monocyte ETs structures.

### Quantitation of Monocyte-Derived ETs

**Figure 3:**
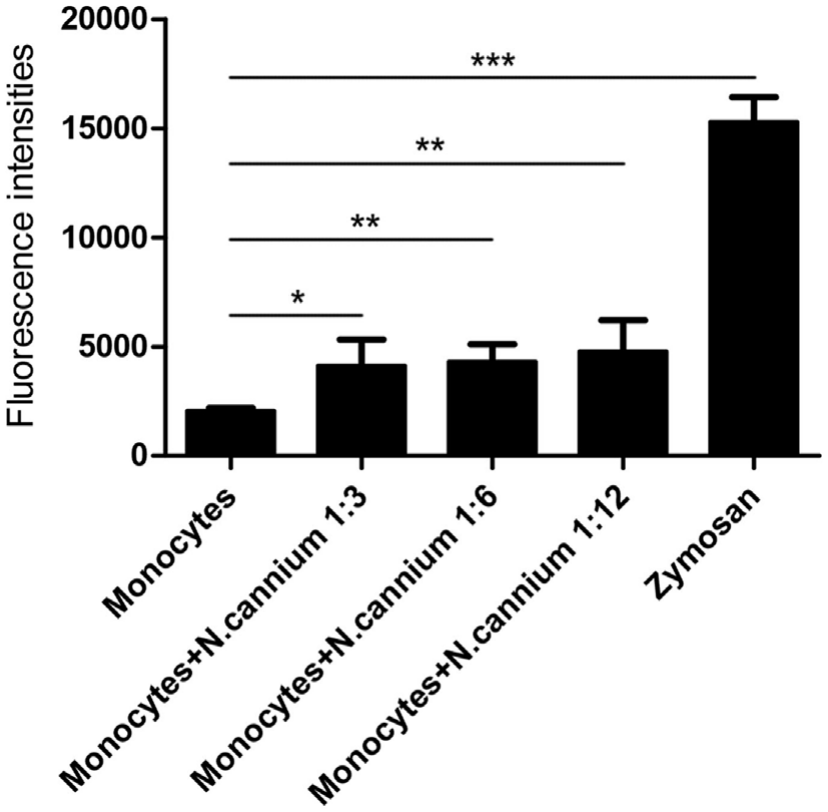
Dose dependency of *Neospora caninum* tachyzoite-triggered extracellular traps (ETs) in caprine monocytes. Caprine monocytes were stimulated with *N. caninum* tachyzoite (monocytes:tachyzoite = 1:3, 1:6, and 1:12) for 60 min. Zymosan (1 mg/ml) was used as positive controls. Adult healthy caprines (*n* = 3) were bleed by puncture of the femoral vein and blood was collected. The formation of caprine monocytes ETs was quantified using Sytox Green, a DNA binding dye. Values are presented as mean ± SD (*n* = 3). *P*-values of <0.05 were considered significant (*P < 0.05, **P < 0.01, and ***P < 0.001).

**Figure 4:**
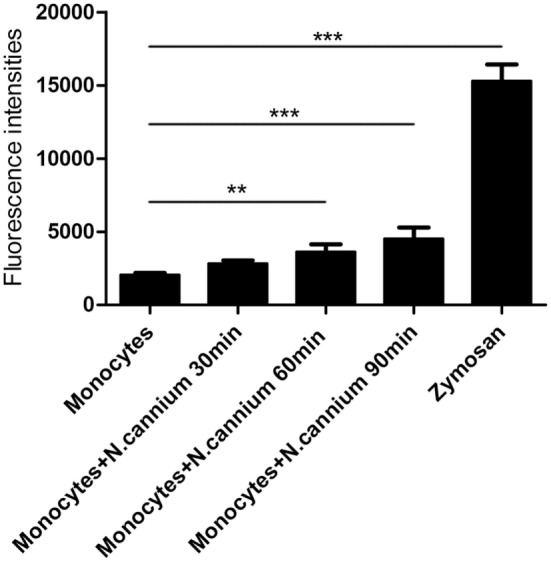
Kinetics of *Neospora caninum* tachyzoite-triggered extracellular traps (ETs) in caprine monocytes. Caprine monocytes were stimulated with *N. caninum* tachyzoite (monocytes:tachyzoite = 1:6) for 30, 60, and 90 min. Zymosan (1 mg/ml) was used as positive controls. Adult healthy caprines (*n* = 3) were bleed by puncture of the femoral vein and blood was collected. The formation of caprine monocytes ETs was quantified using Sytox Green, a DNA binding dye. Values are presented as mean ± SD (*n* = 3). *P*-values of <0.05 were considered significant (**P < 0.01 and ***P < 0.001).

The formation of caprine monocyte-extruded ETs was quantified using Sytox Green, a DNA binding dye. The results of quantitation of monocyte-extruded ETs revealed that *N. caninum* tachyzoites can trigger the formation of ETs in caprine monocytes (Figures 3 and 4). The stimulation with zymosan, which served as positive controls, showed significantly increasing fluorescence intensities when compared with the negative controls, and *N. caninum* tachyzoite-triggered caprine monocyte-ET formation in a dose- and time-dependent process.

### Inhibitors of NADPH Oxidase-, MPO-, ERK 1/2-, and p38 MAPK Signaling Pathway Decreased *N. caninum*-Triggered Caprine Monocyte-ET Formation

**Figure 5:**
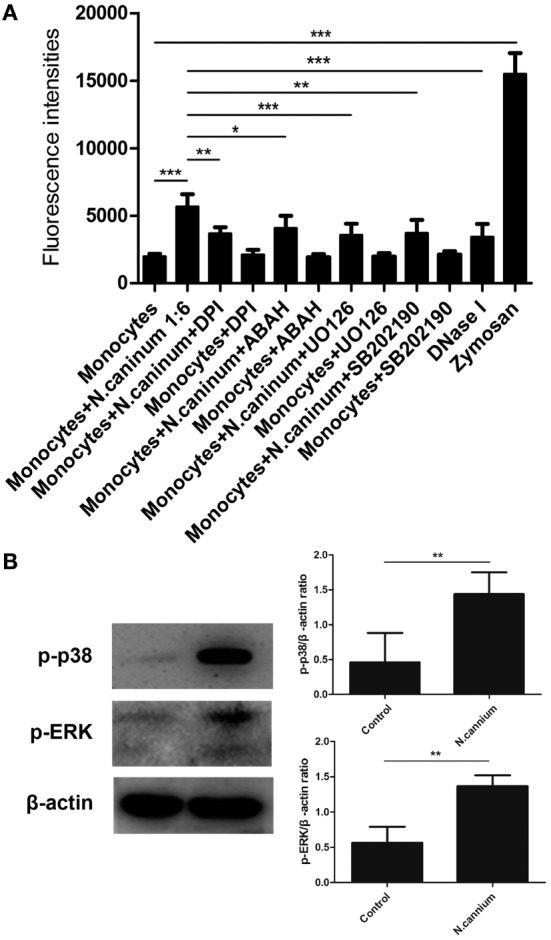
Inhibition of *Neospora caninum* tachyzoite-triggered extracellular traps in caprine monocytes. **(A)** After pretreatment with respective inhibitors: the NADPH inhibitor diphenylene iodonium (DPI, 10 µM), the myeloperoxidase inhibitor (ABAH, 100 μM), the inhibitors of ERK (UO126, 50 µM) and P38 (AB202190, 10 µM) signaling pathway, caprine monocytes were stimulated with *N. caninum* tachyzoite (monocytes:tachyzoite = 1:6) for 90 min. Adult healthy caprines (*n* = 3) were bleed by puncture of the femoral vein and blood was collected. Monocytes stimulated with zymosan (1 mg/ml) were used as positive controls. **(B)** Caprine monocytes were stimulated with *N. caninum* tachyzoite (monocytes:tachyzoite = 1:6) for 45 min. The activities of ERK 1/2- and p38 MAPK signaling pathway were determined by Western blotting. Values are presented as mean ± SD (*n* = 3). *P*-values of <0.05 were considered significant (*P < 0.05, **P < 0.01, and ***P < 0.001).

To investigate the role of these molecules or signaling pathway in *N. caninum*-triggered caprine monocyte-ETs, inhibitors of NADPH oxidase, MPO, ERK, and P38 MAPK were here used in inhibition assays. As shown in Figure 5A, caprine monocytes stimulated with zymosan alone showed significantly enhanced monocyte-ET formation when compared with negative controls. Furthermore, pretreatment of different inhibitors significantly decreased *N. caninum* tachyzoite-triggered caprine monocyte-derived ET formation when compared with monocytes exposed to tachyzoites without inhibition treatments. Furthermore, western blot analysis showed that *N. caninum* significantly increased the activities of ERK 1/2- and p38 MAPK-signaling pathway (Figure 5B), suggesting the role of ERK 1/2- and p38 MAPK-signaling pathway in *N. caninum*-triggered caprine monocyte-ET formation. In addition, *N. caninum* tachyzoite-triggered caprine monocyte-ET formation was significantly inhibited by DNase I treatment, which proved the typical DNA nature in monocyte-released-ETs structures.

### *N. caninum* Tachyzoite Induced ROS Production in the Process of ET Formation

**Figure 6:**
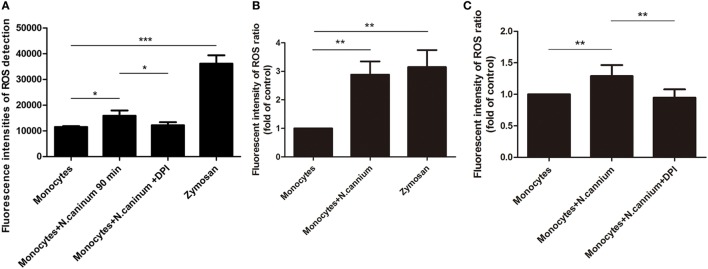
Determination of reactive oxygen species (ROS) in *Neospora caninum* tachyzoite-triggered caprine monocytes extracellular traps formation. Adult healthy caprines (*n* = 3) were bleed by puncture of the femoral vein and blood was collected. Zymosan (1 mg/ml) was used as positive controls. **(A)** Caprine monocytes were stimulated with *N. caninum* tachyzoite (ratio: 1:6) for 180 min. The intracellular ROS production of activated caprine monocytes was examined by a fluorometric plate reader Infiniti M200 at 488 nm excitation/525 nm emission wavelength. **(B,C)** Caprine monocytes were stimulated with *N. caninum* tachyzoites (ratio: 1:3 or 1:6) for 180 min. The intracellular ROS production of activated caprine monocytes was examined by flow cytometry at 488 nm excitation/525 nm emission wavelength. Values are presented as mean ± SD (*n* = 3). *P*-values of <0.05 were considered significant (*P < 0.05 and ***P < 0.001).

The intracellular ROS production of activated caprine monocytes was examined using a fluorometric plate reader Infiniti M200 and flow cytometry. Monocytes stimulated with zymosan showed significantly enhanced monocyte-released ETs, and caprine monocytes exposed to *N. caninum* tachyzoites resulted in significantly enhanced ROS production when compared with negative controls (Figure 6). Furthermore, the monocyte ROS production inhibition through the DPI treatment, which resulted in significantly reduced *N. caninum*-derived monocyte ET formation (Figure 6), clearly confirmed the pivotal role of ROS in caprine monocyte-mediated ETosis.

### Caprine Monocyte-Derived ETs Seemed Not to Be Linked to LDH Activities

**Figure 7:**
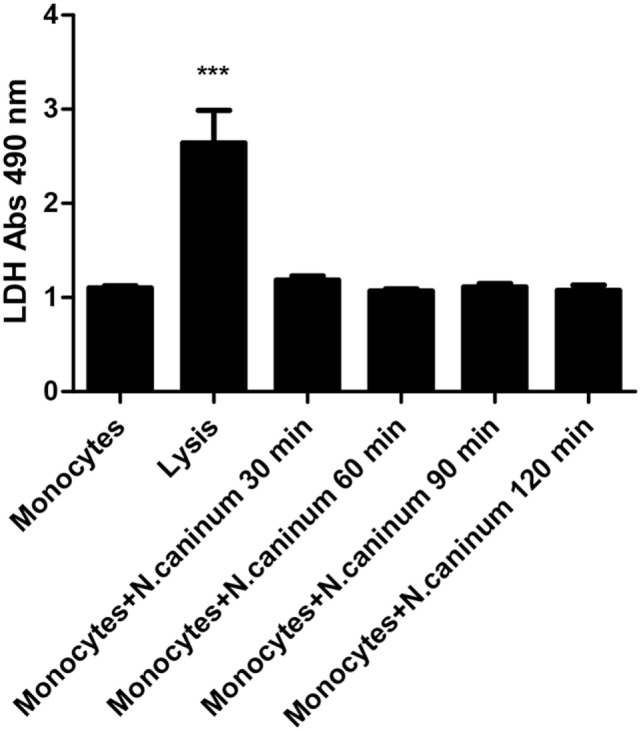
Determination of lactate dehydrogenase (LDH) level in *Neospora caninum* tachyzoite-triggered caprine monocytes extracellular traps formation. Adult healthy caprines (*n* = 3) were bleed by puncture of the femoral vein and blood was collected. Caprine monocytes were stimulated with *N. caninum* tachyzoite (ratio: 1:1) for 30, 60, 90, and 120 min. Values are presented as mean ± SD (*n* = 3). *P*-values of <0.05 were considered significant (***P < 0.001).

In order to prove the importance of LDH activities in monocyte-derived ET formation, the supernatant of caprine monocytes exposed to vital tachyzoites of *N. caninum* was measured by the LDH Cytotoxicity Assay *in vitro*. The results of LDH measurement showed that *N. caninum* tachyzoite-triggered caprine monocyte-derived ETs were not significantly correlated with intracellular monocyte LDH activities (Figure 7).

## Discussion

Neutrophil extracellular traps (NETs) were firstly described as a novel early effector against invasive pathogens in 2004 (20), and this phenomenon was also identified to occur in other leukocyte populations of the innate immune system, such as eosinophils, mast cells, monocytes and macrophages. ETs are composed of DNA fibers and proteins including H3, MPO, elastase and cathelicidins among others (20, 21). These fiber-like extracellular have recently been recognized as a physical barrier and novel mechanism against invasive pathogens, such as the bacteria *Escherichia coli, Staphylococcus aureus*, the fungi *Candida albicans*, and the some apicomplexan parasites, i.e. *T. gondii, Besnoitia besnoiti*, and *Eimeria bovis* (5, 15, 16, 22–24). However, the effect of *N. caninum* on the formation of ETs in caprine monocytes has not been investigated yet, although it is well known that professional phagocytes participate in the immune response and are recruited to the site of *N. caninum*-infected endothelium (25).

This study showed that caprine monocytes cast ETs in response to *N. caninum* tachyzoites, which was similar to bovine PMNs (26) and Bottlenose dolphins neutrophils (27). However, *N. caninum* tachyzoites-induced caprine monocytes-ETs release was not that much as neutrophils, reflecting different cell types playing different roles in *N. caninum* infection. So, factors leading to the difference of ETs release between monocytes and neutrophils need to be explored, and whether ETs release working in conjunction with phagocytosis and degradation in against *N. caninum* infection also need further research. In this study, the results just emphasize the relevance of this novel mechanism in the defense of *N. caninum* as parasite-triggered monocyte-derived ET formation actively interferes with the motility of tachyzoites thereby abrogating their capacity to invade a host cell which is ultimately linked to the obligate intracellular lifecycle as demonstrated elsewhere (15, 28) for other closely related apicomplexan parasites (29, 30). Thus, results of SEM analyses showed that thicker and thinner network structures were released from caprine monocytes after the exposure to *N. caninum*, and *N. caninum* tachyzoites were captured in these structures. The classical ETs mainly consist of chromatin. And we confirm the DNA-nature of tachyzoite-triggered monocyte-derived ETs by staining with Sytox Orange. Additionally, the resolution of tachyzoite-induced ETosis by DNase I treatments proved this typical characteristic of ETs. Alongside chromatin/DNA, the major components of ETs are nuclear H3 and NE, MPO, lactoferrin, pentraxin, and gelatinase among others (13, 31). These classical components are of significant relevance concerning the antiparasitic mechanism of ETs (15, 32). Consistently, applying colocalization analysis concerning extracellular DNA and H3 and MPO in tachyzoite-entrapping structures, we corroborated these classical characteristics of caprine monocyte-derived ETs. Furthermore, MPO inhibitor treatment significantly decreased ET release in tachyzoite-exposed monocytes revealing the essential role of these enzymes in *N. caninum-*triggered ETosis.

The process of parasite-induced ETosis depends on the assembly/activation of the NADPH oxidase (NOX) (15, 29, 30) complex leading to ROS production (33, 34). As reported for several parasites and bacteria, *N. caninum*-induced ETosis also proved to increase ROS production. *N. caninum* tachyzoite stage entrapment in monocyte-ETs proved to be dose and time dependent. Consistently, time- and dose-dependent ET formation has also been previously reported in *T. gondii*- and *E. bovis*-triggered reactions in harbor seal and bovine PMN (15, 24). All these results clearly suggest that *N. caninum* tachyzoites are as well inducers of ETs derived from caprine monocytes.

Monocytes stimulated with zymosan showed significantly enhanced monocyte-derived-ETs formation, thus proving that zymosan is a useful tool for triggering ETs in the caprine system, as previously reported elsewhere (28).

Furthermore, inhibition of MPO *via* its respective inhibitor ABAH, significantly reduced *N. caninum* tachyzoite-triggered fluorescence signals, which suggested the vital role of the enzyme in caprine monocytes-ETs formation. Previous studies have demonstrated that the activation of ERK 1/2 and p38 MAPK signaling pathway was involved in PMA-induced NET formation (35). To further investigate these signaling pathways in *N. caninum* tachyzoite-triggered monocyte ETs, the specific inhibitors UO126 and SB202190 were used in inhibition assays. The inhibition of ERK1/2 and p38 MAPK resulted in significant reduction of *N. caninum* triggered monocyte ET formation and western blotting showed that *N. caninum* significantly increased the activities of ERK 1/2 and p38 MAPK signaling pathway. These results were in accordance to recent investigations on parasite-induced ETosis, such as *T. gondii, Cryptosporidium parvum*, and *Eimeria bovis* (15, 30).

Given that ETosis is considered as a novel cell death process (31), we speculated whether *N. caninum* tachyzoite-triggered ETs formation in caprine monocytes was similar to these characteristics in NETosis. The release of LDH, which is well-known marker of necrosis was not detected during NETosis induced by several stimuli (20, 22, 36). The exposure of *N. caninum* for 30, 60, 90, and 120 min resulted in no LDH activities as the LDH Cytotoxicity Assay did not detect any LDH in the supernatants, thus proving that this process was indeed ETosis. Moreover, these results suggest that *N. caninum*-induced ETosis in caprine monocytes is not correlated with membrane damage, which is in line with the characteristics of NETosis.

In conclusion, this study demonstrates for the first time *N. caninum* as inducers of monocyte-derived ETosis. Furthermore, several molecules as well as signaling pathways involved in *N. caninum* tachyzoite-triggered caprine monocyte-derived-ETosis. However, whether caprine monocyte-ETosis indeed plays a critical role in the early host immune response against this parasite *in vivo* deserves further research.

### Statistical Analysis

Experimental data were analyzed by the GraphPad 5.0 software. The differences among the groups were analyzed by one-way analysis of variance with Tukey multiple comparison test. All values were expressed as the means ± SD. *P*-values <0.05 were considered as statistically significant.

## Ethics Statement

All animal experiments were approved by Laboratory Animal Welfare of Jilin University.

## Author Contributions

ZY, CH, AT, and XZ designed the project and experiments. ZY, ZW, XH, and XW carried out most of the experiments. ZY, ZW, and CH wrote the manuscript. ZW, PG, and JL carried out statistical analysis and prepared figures. JL and XZ corresponded this article. All authors reviewed the manuscript.

## Conflict of Interest Statement

The authors declare that the research was conducted in the absence of any commercial or financial relationships that could be construed as a potential conflict of interest.
